# Supplementary material for: Integrating augmented reality virtual patients into healthcare training: A scoping review of learning design and technical requirements
Source: PLoS One. 2025 Jul 16;20(7):e0324740. doi: 10.1371/journal.pone.0324740 (PMC12266438; doi:10.1371/journal.pone.0324740)
Supplement: S2 File — (DOCX) [file pone.0324740.s002.docx]

**Supporting** **Information table1-** Pub Med OCT 2024

| **Results** | **Query** | **Search** |
| --- | --- | --- |
| **1,704** | ("design"[Title/Abstract]) AND ("virtual patient"[Title/Abstract]) OR ("patient, simulation*"[Title/Abstract]) OR ("simulate, patient*"[Title/Abstract]) | **#1** |
| **6,557** | (("augmented reality"[Title/Abstract]) OR ("mixed reality"[Title/Abstract])) OR ("extended reality"[Title/Abstract]) | **#2** |
| **1,037** | ("design"[Title/Abstract]) AND ("virtual patient"[Title/Abstract]) OR ("patient, simulation*"[Title/Abstract]) OR ("simulate, patient*"[Title/Abstract]) AND ("augmented realit*"[Title/Abstract]) OR ("mixed realit*"[Title/Abstract]) OR ("extended realit*"[Title/Abstract]) NOT ("virtual realit*"[Title/Abstract]) | **#1 AND #2** |
| **99** | #1 AND #2 article type : Case Reports, Classical Article, Clinical Study, Clinical Trial, Clinical Trial Protocol, Controlled Clinical Trial, English Abstract, Guideline, Interactive Tutorial, Interview, Introductory Journal Article, Practice Guideline, Randomized Controlled Trial. | **#3** |
| **69** | #1 AND #2 Species: Humans | **#4** |
| **59** | #1 AND #2 Article language: English | **#5** |
| **23** | #1 AND #2 Text availability: Free full text | **#6** |

**Supporting Information table 2.** science direct OCT 2024

| **Results** | **Query** | **Search** |
| --- | --- | --- |
| **309** | "virtual patient"[Title/Abstract] | **#1** |
| **2,804** | (("augmented reality"[Title/Abstract]) OR ("mixed reality"[Title/Abstract])) OR ("extended reality"[Title/Abstract]) | **#2** |
| **499** | ("design"[Title/Abstract]) AND ("virtual patient"[Title/Abstract]) OR ("patient, simulation"[Title/Abstract]) OR ("simulate, patient"[Title/Abstract]) AND ("augmented realit"[Title/Abstract]) OR ("mixed realit"[Title/Abstract]) OR ("extended realit"[Title/Abstract]) NOT ("virtual realit"[Title/Abstract]) | **#1 AND #2** |
| **193** | #1 AND #2 article type : research article(154), Conference abstract(13), Conference info(1), other(25) | **#3** |
| **151** | #1 AND #2 Subject areas: medicine and dentistry(87), Nursing and health professional(70), Pharmacology and toxicology and pharmaceutical science(25) | **#4** |
| **147** | #1 AND #2 Languages: English | **#5** |
| **24** | #1 AND #2 Access type: Open Access and Open Archive | **#6** |

**Supporting Information table 3.** Cochrane library OCT 2024

| **Results** | **Query** | **Search** |
| --- | --- | --- |
| **Embase: 677**  **CT.gov: 118**  **CINAHL: 75**  **ICTRP: 58**  **928** | ("design"[Title/Abstract]) AND ("virtual patient"[Title/Abstract]) OR ("patient, simulation*"[Title/Abstract]) OR ("simulate, patient*"[Title/Abstract]) | **#1** |
| **Embase: 311**  **CT.gov: 169**  **ICTRP: 108**  **CINAHL:24**  **612** | (("augmented reality"[Title/Abstract]) OR ("mixed reality"[Title/Abstract])) OR ("extended reality"[Title/Abstract]) | **#2** |
| **Embase: 410**  **CT.gov: 71**  **ICTRP: 47**  **CINAHL:12**  **540** | ("design"[Title/Abstract]) AND ("virtual patient"[Title/Abstract]) OR ("patient, simulation*"[Title/Abstract]) OR ("simulate, patient*"[Title/Abstract]) AND ("augmented realit*"[Title/Abstract]) OR ("mixed realit*"[Title/Abstract]) OR ("extended realit*"[Title/Abstract]) NOT ("virtual realit*"[Title/Abstract]) | **#1 AND #2** |
| **540** | - #1 AND #2 article type : Trials(540), NOT Cochrane Reviews(1), NOT Cochrane Protocols(1) | **#3** |

**Supporting Information table 4.** ERIC OCT 2024

| **Results** | **Query** | **Search** |
| --- | --- | --- |
| **2,195** | ("design"[Title/Abstract]) AND ("virtual patient"[Title/Abstract]) OR ("patient, simulation*"[Title/Abstract]) OR ("simulate, patient*"[Title/Abstract]) | **#1** |
| **3,692** | (("augmented reality"[Title/Abstract]) OR ("mixed reality"[Title/Abstract])) OR ("extended reality"[Title/Abstract]) | **#2** |
| **241** | ("design"[Title/Abstract]) AND ("virtual patient"[Title/Abstract]) OR ("patient, simulation*"[Title/Abstract]) OR ("simulate, patient*"[Title/Abstract]) AND ("augmented realit*"[Title/Abstract]) OR ("mixed realit*"[Title/Abstract]) OR ("extended realit*"[Title/Abstract]) NOT ("virtual realit*"[Title/Abstract]) | **#1 AND #2** |
| **41** | - #1 AND #2 Filters: Full text | **#3** |

**Supporting Information table 5.** Web Of Sciences OCT 2024

| **Results** | **Query** | **Search** |
| --- | --- | --- |
| **81,537** | ((((ALL=(“design”)) AND ALL=("Virtual patient")) OR ALL=("patient simulations")) OR ALL=(simulations AND patient)) OR ALL=(patient AND simulation) | **#1** |
| **44,726** | ((ALL=("Augmented reality")) OR ALL=("mixed reality")) OR ALL=( "extended reality") | **#2** |
| **6,122** | ((((((((ALL=(“design”)) AND ALL=("Virtual patient")) OR ALL=("patient simulations")) OR ALL=(simulations AND patient)) OR ALL=(patient AND simulation)) AND ALL=("Augmented reality")) OR ALL=("Mixed reality")) OR ALL=("extended reality")) NOT ALL=("virtual reality") | **#1 AND #2** |
| **2,099** | #1 AND #2 Quick filters: Open Access | **#3** |
| **1,312** | #1 AND #2 document Types: Article(1,309), Early Access(34) | **#4** |
| **1,299** | #1 AND #2 Article language: English | **#5** |
| **324** | #1 AND #2 Web of sciences category: Health Sciences and approaches | **#6** |

**Supporting Information table 6.** Scopus OCT 2024

| **Results** | **Query** | **Search** |
| --- | --- | --- |
| **23,519** | "design" ANA "Virtual Patients" OR "Patient Simulations" OR patient AND simulations OR simulation AND patient | **#1** |
| **63,846** | "Augmented reality" OR "mixed reality" OR "extended reality" | **#2** |
| **89** | "design" ANA "Virtual Patients" OR "Patient Simulations" OR patient AND simulations OR simulation AND patient AND "Augmented reality" OR "mixed reality" OR "extended reality" NOT "virtual reality". | **#1 AND #2** |
| **50** | #1 AND #2 document Types: Article | **#3** |
| **49** | #1 AND #2 Article language: English | **#4** |
| **31** | #1 AND #2 Quick filters: Open Access | **#5** |
