# Supplementary material for: Integrating augmented reality virtual patients into healthcare training: A scoping review of learning design and technical requirements
Source: PLoS One. 2025 Jul 16;20(7):e0324740. doi: 10.1371/journal.pone.0324740 (PMC12266438; doi:10.1371/journal.pone.0324740)
Supplement: Attachment — Submitted filename: Reviewers Report.docx [file pone.0324740.s003.docx]

**Peer Review Report**

Manuscript Title

"Integrating Augmented Reality Virtual Patients into Healthcare Training: A Scoping Review of Learning Design and Technical Requirements"

**Reviewer’s Comments:**

This manuscript addresses a highly relevant and emerging area in healthcare education by exploring the integration of augmented reality with virtual patient technologies; it demonstrates scholarly intent, covers a diverse range of studies, and has strong potential to contribute meaningfully to the development of immersive learning frameworks if revised in alignment with scoping review best practices. I recommend acceptance of this manuscript, provided the authors address the following critical comments.

The authors reference PRISMA-ScR and JBI guidelines and adopt a PCC framework, which is appropriate.

*The manuscript refers to a “systematic scoring process” for evaluating included studies (p.6); however, no validated appraisal tool is cited.* In “*Data collection process”* clarify whether the authors formally appraised the quality of the included studies, and if so, to:

Name the tool used (e.g., JBI checklist),

Explain the process, and

Justify its use in the context of a scoping review.


Best wishes,

Dr Shahzad Ahmad

Senior Lecturer/ Unit Lead

School of Medicine, UOB, Buckingham, UK.
